# Energy-Aware System Design for Autonomous Wireless Sensor Nodes: A Comprehensive Review

**DOI:** 10.3390/s21020548

**Published:** 2021-01-14

**Authors:** Olfa Kanoun, Sonia Bradai, Sabrine Khriji, Ghada Bouattour, Dhouha El Houssaini, Meriam Ben Ammar, Slim Naifar, Ayda Bouhamed, Faouzi Derbel, Christian Viehweger

**Affiliations:** 1 Measurement and Sensor Technology, Technische Universität Chemnitz, Reichenhainer Straße 70, 09126 Chemnitz, Germany; sonia.bradai@etit.tu-chemnitz.de (S.B.); sabrine.kheriji@etit.tu-chemnitz.de (S.K.); ghada.bouattour@etit.tu-chemnitz.de (G.B.); dhouha.el-houssaini@etit.tu-chemnitz.de (D.E.H.); meriam.ben-ammar@etit.tu-chemnitz.de (M.B.A.); slim.naifar@etit.tu-chemnitz.de (S.N.); ayda.bouhamed@etit.tu-chemnitz.de (A.B.); christian.viehweger@etit.tu-chemnitz.de (C.V.); 2 Smart Diagnostic and Online Monitoring, Leipzig University of Applied Sciences, Wächterstrasse 13, 04107 Leipzig, Germany; faouzi.derbel@htwk-leipzig.de

**Keywords:** energy harvesting, hybrid energy harvesting, wireless energy transfer, energy management, wake-up receiver, energy saving, energy efficiency, energy prediction, wireless sensor networks, compressive sensing

## Abstract

Nowadays, wireless sensor networks are becoming increasingly important in several sectors including industry, transportation, environment and medicine. This trend is reinforced by the spread of Internet of Things (IoT) technologies in almost all sectors. Autonomous energy supply is thereby an essential aspect as it decides the flexible positioning and easy maintenance, which are decisive for the acceptance of this technology, its wide use and sustainability. Significant improvements made in the last years have shown interesting possibilities for realizing energy-aware wireless sensor nodes (WSNs) by designing manifold and highly efficient energy converters and reducing energy consumption of hardware, software and communication protocols. Using only a few of these techniques or focusing on only one aspect is not sufficient to realize practicable and market relevant solutions. This paper therefore provides a comprehensive review on system design for battery-free and energy-aware WSN, making use of ambient energy or wireless energy transmission. It addresses energy supply strategies and gives a deep insight in energy management methods as well as possibilities for energy saving on node and network level. The aim therefore is to provide deep insight into system design and increase awareness of suitable techniques for realizing battery-free and energy-aware wireless sensor nodes.

## 1. Introduction

Wireless sensor networks are becoming increasingly important, supported by several trends of digitalization [1,2,3]. Different trends towards the Internet of Things (IoT), Industry 4.0 and 5G networks address massive sensing and implicitly admit to have wireless sensors delivering measurement data directly to the web in a reliable and easy manner. Therefore, wireless communication alone is not sufficient. An autonomous energy supply is also decisive for flexible positioning and easy maintenance. Both aspects are important for the acceptance of wireless sensors and consequently for realizing a massive use [4].

Recent developments have revealed interesting possibilities to enhance energy harvesting (EH) efficiency by the design of dedicated converters [5,6,7], combination of converters in hybrid solutions [7] and adopting possibilities for wireless energy transmission [8,9]. Developments of microelectronics enable significant energy savings, so that energy supply from ambient sources becomes increasingly practicable [10,11]. Wake-up receivers allow fully switching off unnecessary system parts and significantly reducing the energy consumption during sleeping phases [12]. Data aggregation techniques, clustering and intelligent routing enable significant energy savings on network level [13]. Supported by the diversity of individual techniques on node and network levels and by a suitable system design implementing novel techniques, optimized energy autonomous wireless sensors can be realized with an outstanding acceptability.

Several interesting reviews give insight into the field of energy harvesting concerning materials and technology [14,15,16] or a certain type of an ambient source, such as photovoltaic [17], piezoelectric converters [18], thermoelectric converters [19] and triboelectric converters [20,21]. Other reviews focus on the use of energy harvesting from the point of view of a certain application field such as the industrial [22], medical [23,24] or environmental field [19]. Another category of reviews deals with individual technologies and sub-modules of energy harvesting supplied wireless sensors, such as hybridization [25,26], energy management [27,28], wake-up technologies [29] or wireless communication aspect [30] or provide a global view of energy harvesting [31] for elaborating prospects for future developments.

In this paper, we aim to provide an overview of the system design of energy autonomous wireless sensors. After a review of the challenges in the design of energy autonomous wireless sensors in Section 2, we recapitulate possible supply strategies in Section 3, where we give an overview of energy harvesting possibilities and focus on hybrid converters using multi-sources as well as wireless power transfer as an interesting supplement to ambient sources. In Section 4, we highlight different aspects of energy management including possibilities to maximize energy income, combine energy sources and reduce energy consumption at the level of the wireless node. We explicitly treat possible operating modes depending on energy availability and how to optimize the starting phase of wireless sensors from zero energy conditions. In Section 5, we consider possibilities for energy saving on the network level and give an overview of the most relevant approaches. In Section 6, we summarize the main results and give some future perspectives.

## 2. Challenges in System Design of Energy Autonomous Wireless Sensors

**Figure 1:**
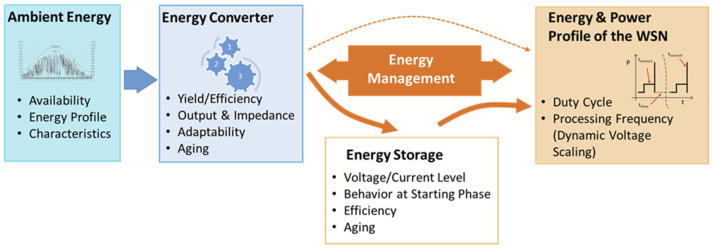
Challenges for the supply of WSNs with energy harvesting.

One of the most important challenges in the design of WSN is to ensure sustainable operation of nodes while maintaining a good connectivity and network longevity. For a supply by ambient energy, the profile of the ambient source needs to be considered for deciding about suitable energy converters, optimizing their parameters and size and assessing the expected total energy (see Figure 1). Thereby, not only the amount of energy is important, but also the energy availability and how it is distributed and fluctuating over time, the so-called energy profile.

Energy converters need to realize a good yield and a high conversion efficiency where they are subjected to changes of environmental conditions and unavoidable aging effects. As application requirements need to be fulfilled along operation life; only improving energy efficiency of harvesters or using an energy storage unit with a higher capacity is not sufficient. The energy management should fulfill a stable energy availability along the lifetime despite fluctuating, non-stable and not available timely energy income, so that the sensor system operation is not affected by changes of environmental conditions or along time periods with a low energy availability. The energy source may be not sufficiently available or may not be reliably available all the time, so that solutions need be searched to cover these limits.

The sensor system needs to be designed for low-power consumption and have different operation modes which can be selected depending on the energy availability, so that, if the energy is no longer sufficient for the operation mode with the lowest energy consumption, the WSN can start automatically and come back to operation mode once it obtains enough energy form ambient sources.

All these aspects are strongly related to energy availability and system design of the wireless sensor node and sensor network. It is therefore important to take care of them during the design of all components and modules in wireless sensors by efficiency improving the energy converters, energy management and energy-aware communication in WSNs. The design process is therefore interdisciplinary and needs special care to realize practicable systems and fulfill the needs of the application in an optimal manner.

## 3. Energy Supply for Wireless Sensor Nodes

**Figure 2:**
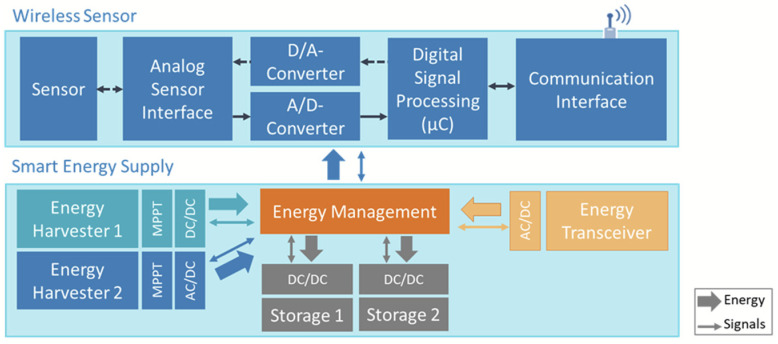
Generalized energy supply strategies of wireless sensor nodes based on energy harvesting, hybrid energy harvesting and wireless energy transfer.

Harvesting energy from ambient sources is challenging since it is limited, unstable, random and variable relative to the application and the environmental condition. To enhance the available energy for wireless sensor networks, several techniques have been proposed to improve the performance of energy autonomous systems from real ambient sources [1]. Nevertheless, both ambient sources and converters have their own limitations. Because the design of the converter is decisive for enough energy yield, the first strategy for improving system performance is the converter design, which should be fitted to the energy source by considering its characteristics (see Figure 2). A further strategy for improvement of the energy yield and realizing a more reliable energy availability is the use of more converters and the development of hybrid converters harvesting energy from the same source in different ways or harvesting energy from multiple sources (see Figure 2). The same applies to energy saving, which could be strategically divided into several storage units allowing a faster availability in restarting phases and a better power density, i.e., the energy per time, especially for the node communication.

In several applications, the availability of the measured data should be guaranteed even if ambient energy sources are not sufficient. In these cases, but also in general, supplying wireless sensors by energy transfer is an interesting possibility to couple energy to the system in a dedicated manner. It is also interesting to provide energy in the case of sudden or unexpected changes of environmental conditions as well as in the case of components aging in a way that could not be considered within the system performance assessment in the system design process. All these strategies are detailed in the next sections.

### 3.1. Overview of Energy Converters

Harvesting from ambient sources is, despite the manifold harvesting principles and technologies available, still challenging due to the limited generated energy and instability of the sources depending on the application and the environmental conditions. To enhance the available energy for WSNs, several studies has been conducted aiming to improve the performance of energy harvesting systems considering real ambient sources [4]. Thereby, each ambient source and converting principle has its own characteristic and power density level. Table 1 presents a summary of the most common existing ambient sources including solar, wind, vibration, thermal, ocean waves, nuclear reaction, acoustic noise and radio frequency (RF) energy. The highest energy income can be realized from solar cells outdoors (see Table 1). This possibility remains the most predictable as weather data can be used as a priori knowledge in energy management [32]. Mechanical energy is also remarkably interesting as it provides possibilities to adapt the harvester to the source and extract a high level of energy [33]. It is also interesting from the point of view of availability, e.g., in environmental and industrial applications. An ambient energy source such as vibration can be found in several applications, such as in industrial field in machines, transport, infrastructure such as bridges and human movements for wearable applications. The wind source [20] is widely available in the environment but the challenges for harvesting energy are often the weak wind, maintenance cost and the strong fluctuation. Further, as source, we can find the ocean wave energy, which is considered as the largest estimated global resource form of ocean energy, and it is useful for several applications such as electricity generation, water desalination and pumping of water [34].Acoustic noise is well known as a source for energy harvesting in industrial plants, transport (e.g., airplanes, vehicles and high-speed trains) and loudspeakers. Thermal energy harvesters [19] have a relatively weak energy income at 40 µW/cm^3^ and are therefore interesting if they can be applied with a big surface or at high temperature differences in certain environments.

To supply wireless sensors, the availability of the energy source and its properties are important. They should be considered in the design to maximize the energy income and increase efficiency. The harvester needs to be scaled depending on the expected energy level and the necessary supply level. To compensate energy fluctuation and, especially depending on the length of time intervals, where the energy income is inferior to the energy consumption, an energy storage unit needs to be selected and dimensioned, as explained in Section 4.

### 3.2. Hybrid Converters

A strategy for increasing energy income and more energy reliability for a WSN is the development of hybrid converters harvesting energy from multiple sources. In hybrid solutions, more converters from the same source or combinations of harvesters from different energy sources can be considered. A good synergy between harvesters can be realized, if the sources are relatively independent and can be available in the same environment. In the case of hybrid converters, the amount of harvested energy compared to the single source is of great interest. Hybrid solutions therefore aim to have a compromise between improving the generated energy and the improvement of the reliability of the harvesting system. That is why the hybrid solution is used more often where several ambient sources coexist, which can be complementary and ensure a better reliability. If they have a different behavior under operation conditions, they may get more complementarity, so one source could take over in phases, where the other lacks energy from ambient. In addition, the dependence on environmental conditions and aging may differ, bringing a better total system availability over the lifetime. In general, it is not necessary that the harvesters contribute with the same amount of energy. The challenge is more to increase the total energy income and the availability of the system under the different operation and environmental conditions as well as independently of aging.

**Figure 3:**
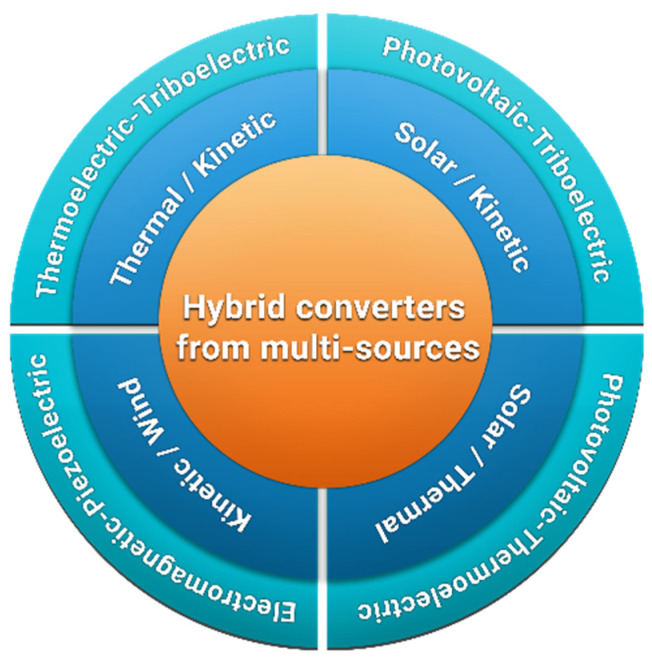
Overview of selected hybrid converters from multi-sources.

Often reported hybridizations consist of the development of multi-converters harvesting from solar source combined with thermal or kinetic source, the thermal source in combination with the kinetic source and the kinetic source combined with the wind source. Figure 3 provides an overview of interesting combinations of ambient sources reported in literature.

**Figure 4:**
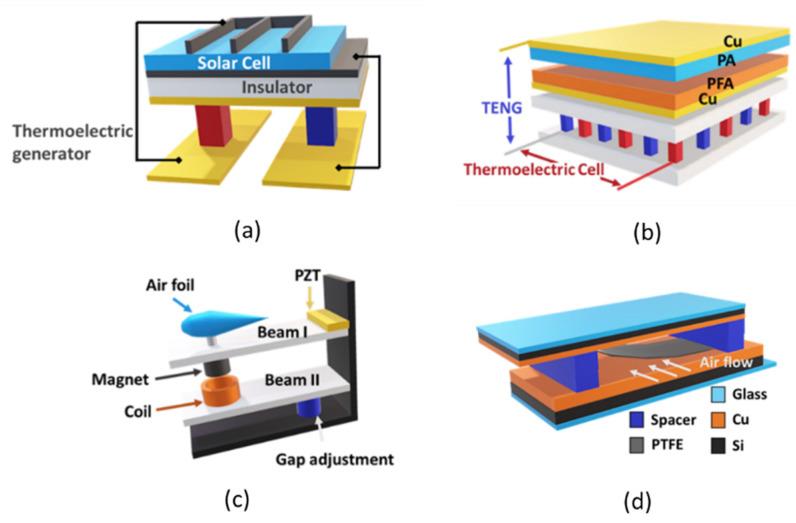
Selected examples of hybrid converters including: (**a**) photovoltaic/thermoelectric generator (see [41]); (**b**) triboelectric/thermoelectric generator (see [42]); (**c**) piezoelectric/electromagnetic generator (see [43]); and (**d**) photovoltaic/triboelectric generator (see [44]).

For the realization of converters combining thermal and solar sources (see Figure 4a), a laminated structure is proposed in [36,37], where solar and thermoelectric cells are used. This hybridization enabled in this case an improvement of the maximum power output by 47% and 36% in comparison to the single solar cell and thermoelectric cell, respectively. The resulting system structure is complex and contains several layers to be able to ensure the hybridization. In [37,38], a solution combining thermoelectric and solar cells is developed to power wearable medical devices. In this case, the main purpose of combining both sources is to ensure the continuous reliable energy availability as photovoltaic cells have a good performance limited to outdoors. Thermal source is added, in this case, especially to compensate the gap in cold environment and at night, where solar cells have a low performance or even cannot generate energy. In [39], an interesting solution, where one single cell combining intrinsically thermoelectric and photovoltaic effects is proposed and showed an efficiency improvement by 23.3%.

In [40,41], the combination of solar energy with kinetic energy is investigated. In this case, the kinetic source consists of a raindrop harvester aiming to improve the performance of solar cells during rain by using the triboelectric effect. The hybridization is in this case limited to the presence of rain. Other intermediate conditions such as cloudy or foggy days are not considered.

Another solution, combining triboelectric principle, thermoelectric principle and solar cells together under a single structure (see Figure 4b) is proposed in [23]. It can harvest simultaneously or individually mechanical, thermal and solar energies. It is a promising solution in the future especially for applications with variable operating conditions.

Aiming to harvest from vibration and wind sources simultaneously, the authors of [43] developed converters based on electromagnetic and piezoelectric principles (see Figure 4c,d) able to harvest energy from ambient vibration of bridges. The results show that the energy generated by the wind source is extremely limited and reaches only up to few µW, but the total energy can reach the level of mW if it is combined with the vibration source. Rarely, the thermoelectric source coexists with kinetic source in the case of friction. In this case, the energy is generated by combining thermoelectric and triboelectric effect, which is addressed in [45], where an improvement of 13% is reached compared to single triboelectric effect.

Hybrid converters can principally use also the same ambient source. In [7], a vibration converter is developed combining an electromagnetic converter with a magnetoelectric converter. As both converters use the same mass–spring systems and magnetic field variation generated by a magnetic spring, the energy density could be improved to reach 0.11 mW/cm^3^. Both harvesters have been combined in a synergetic manner and reach together 136% of the sum of energy from both harvesters if they were used alone.

Combining two different energy converters is critical from the economical point of view as more converters are needed together with the corresponding energy management circuits. It necessitates an integration concept for the harvesters and the energy management.

### 3.3. Wireless Power Transfer

Wireless power transfer (WPT) provides several benefits such as safety, flexibility and the possibilities of range extension and battery-free realization. It has a great potential in various applications, such as portable systems, medical systems, non-accessible systems and mobile systems and robots. In energy-aware wireless sensors, various combinations with energy harvesting and battery-free systems can be realized, where the WPT is the main source of energy supply. It can also be used on-demand or permanently as a supplement to energy harvesting. In applications where a high reliability is required, the WPT can provide the functional guarantee. It can therefore promote the spread of sensor systems that are supplied with energy harvesting.

The WPT can be classified by two major types of techniques: far-field transmission and near-field transmission [46]. Far-field transmission realizes, due to safety issues, a low-power transmission level and is therefore interesting in cases where transmission efficiency is not the highest priority. One example for far-field transmission is laser-based transmission through a direct line-of-sight path or microwave transmission at high frequencies. More interesting is the radio frequency transfer [47,48] and capacitive power transfer [49]. Charging via radio frequency offers flexibility of positioning and possibilities of charging in several frequency bands in the range of MHz to GHz. Nevertheless, the main challenge of RF energy transmission is the low power level and the RF-DC power conversion efficiency (PCE). In [48], a passive RF-DC converter is designed for energy harvesting at ultra-low input power at 868 MHz. The converter consists of a reactive matching circuit to minimize discontinuities and signal reflections, a passive Dickson voltage multiplier to increase the voltage level and a rectifier [50]. It stores energy in an input inductor and capacitance during the negative wave and conveys it to the output capacitance during the positive wave. The realized PCE of the proposed RF-DC converter outperforms [48] state-of-the-art RF converters especially at a low input power of −40 dBm.

Near-field WPT realizes a high efficiency and is more practicable considering safety and power level. It can be realized by various methods such as capacitive power transfer (CPT) and inductive power transfer (IPT). In CPT, the sender and the receiver are the plates of an air capacitor [49]. The level of transmitted power is strongly dependent on the distance between the plates. The efficiency becomes extremely low at higher distance. Generally, big plates, having an ideal alignment at extremely low distances, are used to reach an acceptable energy level for supplying a wireless sensor node. Additionally, the dielectric properties of the materials between the two plates, as well as in the environment corresponding to the stray field, have an important influence on the transmission efficiency. This method is therefore not suitable for use in dirty or wet environments [46]. Due to the use of large plates, this technique is not suitable for small devices or ultra-high-power applications [46].

Magnetic Resonance (MR) is a method of wireless power transfer suitable for a medium distance range between sender and receiver, from centimeters to few meters, realizing a medium efficiency [46] and able to transmit power in the range of mW to few W. It is characterized by a high-quality factor due to the self-resonating of the coil between their internal capacitor and inductor in the range of MHz [47,51]. Besides, it can supply multiple devices [51,52] at the same time on different positions. For that, tuning resonance and avoiding interference due to multiple receivers are required during the charging process. To increase the charging distance, some designs introduce resonator coils, which are associated to each side [53].

**Figure 5:**
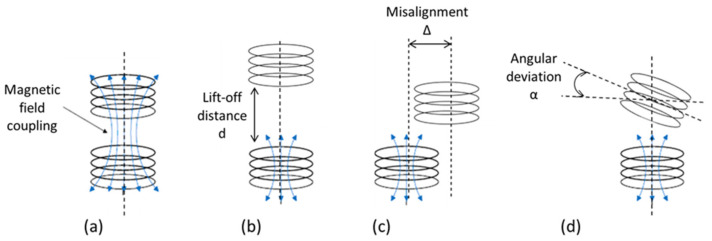
Possible coils positions: (**a**) ideal case; (**b**) big lift-off distance; (**c**) lateral misalignment; and (**d**) angular misalignment.

If the distance between sender and receiver is in the range of few cm or at least smaller than the coil diameter [54], IPT can be used to realize a transmission efficiency superior to 60%. IPT systems transmit power through coils with a specific frequency in the range of hundreds of kHz [46]. Over the last years, significant investigations have been conducted to improve the performance of IPT systems, especially for wireless charging of mobile devices, but also medical applications [55] and electric vehicles [56], which can be scaled up or down for wireless sensors. Different aspects could be optimized considering coil geometry and technology, operating frequency, multi-coil systems, resonant compensation topologies and misalignment. The aspect of misalignment is very important for efficiency of power transfer. An IPT system reaches its best performance under resonance conditions and at concentric transmitter and receiver coil positions with minimal distance to avoid field losses. However, during use, misalignments between sending and receiving coils can occur, which can be lateral, vertical and angular (see Figure 5). These have a negative impact on the level of transmitted power and therefore on transmission efficiency and charging time.

Increasing the tolerance to coils misalignment is one of the main aspects that should be considered in the design of a wireless sensor node or device. For that, a single transmitter-to-single receiver (SISO) coil system should be controlled to increase the transmission efficiency despite variable loads and misalignment. This can be realized by controlling the system frequency [57], the system gain [58], the compensation capacitors [59] and the DC-DC rectifier [60]. To overcome misalignment effects, multiple input–single output (MISO) coil systems can be realized [61], which are composed of multiple transmitter coils connected in series [62,63] or parallel [64,65]. The sending coils can be activated simultaneously [62] or one-by-one independently [65] using multiple supply circuits [66], switches [64,67] or both [68]. The main difference between systems used to charge batteries and those to supply battery-free devices is the detection procedure. In fact, battery-free systems do not use communication for the activation of the transmitter side system due to lack of stored energy, which can be useful to activate communication procedure [65]. However, keeping the transmitter side ON to detect the presence of device for long times increases the energy losses. In [65], the authors proposed a passive peak detector circuit of the receiver coil with controls the working circuit frequency to reduce the power consumption. When the device is detected, the system increases the working frequency to reach the resonance, which helps the system to deliver energy able to supply the circuit of the receiver side to communicate with the transmitter side.

## 4. Energy Management

Energy management is a key component of a system supplied by energy harvesting. An efficient energy management solution enables long-lasting sensor nodes and WSNs powered by ambient energy harvesting. It facilitates the creation of totally maintenance free nodes, which can be, e.g., deployed in harsh environments. 

### 4.1. Energy Budget Estimation and Benchmarking

The energy constraint is a challenging feature for WSNs. Generally, sensor nodes consume energy while sensing, processing, transmitting, or receiving data. This can be defined as useful power consumption. As indicated in [69], more than 50% of energy is devoted to the radio part. The increasing demand for computing and memory, maintaining connectivity, meeting design constraints, low power consumption, low cost and real-time constraints on WSNs applications are leading to the development of novel approaches for efficient hardware architectures. A significant challenge is to design applications with high computing potential with limited resources. To enable the end-to-end performance, the processing complexity is raised at the end nodes, leading to an excessive power consumption that constrains their large-scale deployment. Thus, a compromise between the processing performance and energy consumption needs to be considered. The choice of an energy-efficient microcontroller is crucial in the design of any wireless sensor node. In this context, a benchmarking-based investigation on energy efficiency of low-power microcontroller for WSNs is developed to select the appropriate MCU [70]. The benchmarking presents a method for the characterization of power consumption of microcontrollers to satisfy the performance requirements. The method is based on data sheet comparison and deep comparison, namely real measurements of power consumption in the selected microcontrollers. The power consumption of various microcontrollers in different operation modes for potential use in applications with low-power requirements are analyzed. Considering the data logging operation, the power consumption of the PIC16LF, MSP430FR and STM32L microcontrollers are, respectively, 1.52, 0.965 and 1.135 mW.

Different techniques are implemented to reduce the power consumption of the processing unit of a wireless sensor node including the Dynamic Voltage and Frequency Scaling (DVFS) [71]. The DVFS is designed to minimize the power consumption during the execution of tasks by adjusting dynamically the operating voltage and frequency depending on the system performance requirement at a fixed time. Once the MCU is slightly charged, the frequency and voltage are gradually reduced to ensure that the MCU runtime latency is not affected. The MCU running at maximum frequency is loaded beyond a certain threshold. Therefore, investigating the power consumption of the wireless node during different activities is critical. In fact, a sensor node can have various operating modes, where, in each state, the MCU consumes a certain amount of energy, dependent on the executed function. Five main activity modes are identified for each wireless sensor node: off, idle, sleep, transmission and reception and processing. When the node is turned off, the microcontroller still idles but consumes a minimal amount of energy compared to other modes, which allows it to switch to sleep mode when necessary. Once the node is turned on, it goes to sleep mode to save energy and waits for the next step according to its predefined schedule. When the node is only listening, idle mode is activated. Next is the complete activity mode, where the node is either a receiver or transmitter, which in both cases consumes more energy than previous stages. The energy consumption in each step is relative to the electronic components of the node itself. Table 2 presents an overview of current consumption of wireless sensor nodes of well-known platforms in different activity modes. A precise estimation of the energy consumption within each activity mode is related to the transmitted payload, mainly during reception and transmission modes. Profiling the energy dissipation of the wireless sensor node during its respective activity and operation modes helps to recover its energy and balance the complete energy within the network. This helps to efficiently, manage the energy consumption with respect to the available energy budget and nodes requirements.

### 4.2. Control of Operation Modes

In contrast to classical or battery powered systems, the operation of wireless sensor systems using energy harvesting is usually limited by the amount of harvested energy. In most cases, the limitation of the available energy prohibits continuous and full system function all the time. Instead, power and energy consumption must be distributed well over time by defining several system operation modes and controlling them depending on the energy and power balance. This means, for example:

- Store energy if the input is high, e.g., in solar supplied systems to bridge the night
- Use energy only if really needed, e.g., do not send data if the value has not changed
- Reduce the standby current as much as possible
- Switch off unnecessary parts

**Figure 6:**
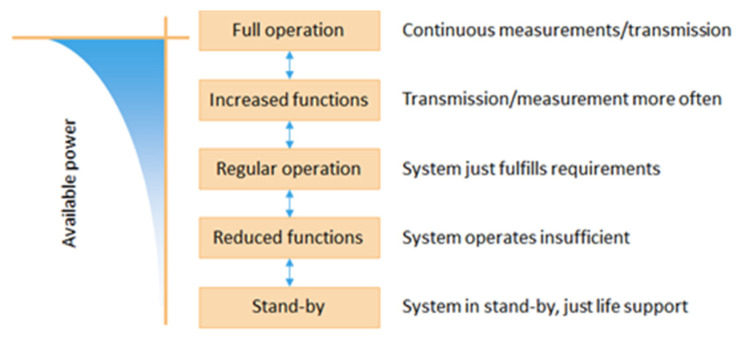
Operation states of a WSN with respect to energy.

Figure 6 gives an overview of the general classification of operation modes. Every sensor system has its specification, e.g., one measurement and one data transmission per time unit. If this functionality can be fulfilled, it can be described as a “regular operation”. If the available energy is lower than required for the “regular operation”, the functionality must be reduced. This means that less than one measurement/transmission per time unit is possible, i.e., the system will work, but the performance is less than expected. This would be a disadvantage and might even cause problems if the gap between two measurements is too long for the application.

If the available energy is not sufficient to acquire and send any data, the system will stay in standby. Usually, in this state of operation, it can still convert and store energy, so that eventually at least an emergency signal can be sent. In some cases, for example for a system supplied by solar energy, there are peaks of incoming power. In these cases, situations might occur where the energy storage elements are already filled and the available income still surpasses the “regular operation” and even the “increased functionality” by far. This state as well as the standby state are unpreferred and can be handled by the dimensioning of the energy storage part of the system or using more storage units. The usual goal of the system design is to reach the “regular operation” state most of the time and usually only deviate with reduced or increased (preferred) operation. The change from one operating mode to another is triggered by conditions and thresholds related to the specification of the sensor node, its typical duty cycle and the expected energy consumption for every subtask as well as the actual available energy in the system.

### 4.3. Energy Management for Single Sources

**Figure 7:**
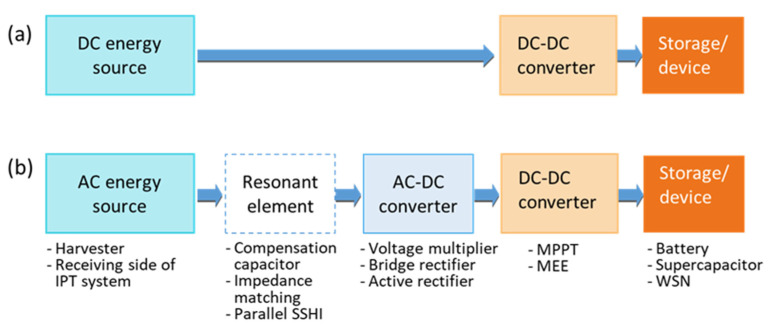
General structure of energy management for (**a**) DC; and (**b**) AC sources.

Energy management circuits generally include several parts, such as impedance matching circuits, rectifiers, voltage multipliers, DC-DC converters or AC-DC converters, to realize the required output level to the load, which can be a battery, a super-capacitor or the WSN itself (see Figure 7). The elements of energy management can also be active, partly passive [76] or completely passive, where the passive solutions do not require energy supply. For example, DC-DC converters are preferred to classic voltage regulators when the working conditions are stable. However, the selection of an optimum regulator is critical when temperature changes and internal losses occur.

Energy harvesting sources have a specific current/voltage behavior and a typically fluctuating energy profile. Energy sources, such as solar cells, may deliver different currents depending on the voltage following their characteristics. A maximum efficiency extraction (MEE) is important, and therefore DC-DC converters or AC-DC converters are used to match the impedance of the source to the connected load impedance. They can thereby realize a voltage multiplication to enhance the level of the output voltage. The main challenge in the design of DC/AC-DC converters is the power consumption due to losses of the diode bridges and switching circuits, which becomes more critical at low power levels. Besides, additional sensors may be required to measure characteristics of the source such as energy, voltage peak level or impedance.

The sources are subject to changes due to environmental conditions and aging. Therefore, maximum power point tracking (MPPT) is needed to follow these changes and maintain an efficient energy extraction. Many control algorithms can be implemented for that, such as perturb and observe, PI controllers, fuzzy logic, neural networks and artificial intelligence algorithm. Tracking maximum power within proper efficiency leads to compensation of changes and environment losses even at a varied duty cycle. MPPT can be realized within AC and DC converters by applying control algorithms to them in a defined manner. Different techniques can be adopted for this, including resistive matching and conjugate impedance matching, depending on the nature of the input of the power processing circuit. Several methods have been demonstrated, such as perturb and observe (P&O) or regulating he output voltage or output current of the diode bridge [77].

Other techniques allow the use of MPPT in certain ambient power scenarios to extract energy even more efficiently, e.g., parallel synchronized switch harvesting on inductor (P-SSHI) [78] and double synchronized switch harvesting (DSSH) [78]. The aim is to synchronize the voltage and current as well as eliminate the phase shift between them to reach a high energy level. The synchronization can be realized electronically, e.g., by implementing peak detectors. In [79], a random mechanical switching harvester on inductor (RMSHI) has been proposed for piezoelectric transducers, which realizes this synchronization directly from the mechanical part; thus, no electric energy is needed for the synchronization, bringing big advantages in the case of ultra-low-energy sources.

### 4.4. Hybrid Energy Management for Multiple Harvesters

Hybrid energy management techniques are developed depending on the properties of the hybrid sources and the application requirements [80]. In [81], an effective technique of harvesting the energy coming from multiple sources is realized through a diode based “Power ORing” architecture. This modular approach collects energy from an arbitrary number of connected harvesting subsystems in a concurrent and independent way. The presence of a diode at the output of each subsystem prevents the exchange of energy between these different harvesting sources, which makes each of them completely independent from the others. They are also able to independently perform MPPT of the transducers, leading to an important increase in the tracking efficiency for each harvester source. A self-synchronized operation is also ensured by using the diodes in this technique, which reduces the complexity of the power management unit (PMU). However, an extra power loss is caused by the forward voltage drop of the diodes.

In [82], a complementary use of energy sources approach is implemented. This technique mainly extracts energy from a thermal generator as a first source to power the active interface of the circuit and then uses a piezoelectric converter as a second source as an energy supply for the auxiliary circuits in the PMU. In the case of insufficient energy income, the cold start-up of the system is ensured by a passive circuit. One limitation mentioned in [82] is that this approach does not combine the energy coming from the hybrid sources to deliver it to the load because only the thermal generator in this case is used as the main power supply for the load circuit while the piezoelectric transducer is applied to charge a small capacitor to provide the supply voltage for some bias and reference circuits.

Another technique is implemented in [83], named shared-inductor DC-DC converters, where buck-boost converters are used to realize impedance matching for MPPT purposes. The hybrid energy sources are assembled, by considering their types, and then combined using a buck-boost based energy combiner technique. Thereby, they all share the same inductor, which reduces the number of components required. The complex controller in this topology must give all the inputs access to the single inductor, while at the same time ensuring that a maximum power transfer for each one of them is reached.

None of the above energy management techniques achieve a highly efficient combination of the hybrid energy sources extracted from each input because the energy coming from the different sources is not combined to be delivered to the load circuit. Therefore, the authors of [84] proposed an approach to overcome this limitation, named energy combining through linear regulators. All the energy extracted from the hybrid sources are thereby rectified separately one by one depending on the electric properties of the source, and then combined by connecting the output of individual linear regulators. An external capacitor is used as a single storage device to save the energy. In [84,85], an effective energy management combining hybrid sources is proposed. Firstly, the energy sources are rectified one by one with the suitable rectifier fitting to the source properties, and then they are combined using operational amplifier circuits for adding or multiplying the voltages. The input sources are connected to a non-inverting op-amp. The purpose is to obtain a non-inverted wave on the output voltage across the resistor. Furthermore, the output of op-amp is directly connected to the designed boost converter, which raises the low voltage using a MOSFET.

Hybrid energy harvesting is a key issue for improving energy availability and reliability to ensure a disturbance free operation of sensor nodes. Nevertheless, the efficiency of energy management needs to be considered. Especially the complexity and energy consumption of the energy management circuit should still maintain the energetic and economic requirements, even though completely different source characteristics need to be considered.

### 4.5. Voltage Supervisor for Cold Start

Energy fluctuations and extreme low availability of ambient energy may lead to a total switch-off of a wireless sensor node. Most current wireless sensor nodes are not able to recover automatically by themselves from a depleted state in a so-called cold start. This is because the energy management requires a minimal energy level to drive a microcontroller-based system, which is not always available once the energy supply reaches a defined level of voltage. This leads to an oscillating behavior of the voltage supply, where the system tries to start, but does not succeed, as the needed energy for starting can be much more than the operation energy needed later.

To ensure the wireless sensor node recovery in these cases, so-called voltage supervisors, voltage detectors or reset integrated circuits can be implemented. Other terms are used in the literature such as buffer circuit [86], control circuit [87], start-up circuit [88,89,90,91], switching circuit [92], trigger circuit [93,94,95] and under-voltage lockout circuit [96]. Commercial voltage supervisors, such as TS831 [97], trigger the reset pin of the microcontroller, as it consumes much more power in the reset state than the active mode. For example, the average consumption of a benchmarking program on a microcontroller (MSPEXP430FR6989) at 3 V is approximately 100 μA, but, during a reset with the same voltage input, it consumes more than 19.8 mA, which is 198 times more and is therefore critical for the energy budget [98]. Several voltage supervisors guarantee the operation only beginning from a certain voltage threshold, which is often around 1 V or more. At lower voltages, the state is undefined, leading to half opened MOSFETs, active loads or oscillating behavior and unwanted power dissipation. Such voltage supervisors are not suitable for typical cold start problems, at low voltage levels near 0 V, which could be a necessity for distributed wireless sensors supplied by energy harvesting from ambient, especially for non-critical applications.

In [89], a hybrid harvester setup is proposed, where the secondary harvester and a passive extraction circuit provide the start-up energy for the control of the main extraction stage. The circuit is relatively complex and the energy for start-up is extracted from a piezoelectric transducer at low efficiency. This setup provides cold-start ability, but it does not treat the power consumption of the load. Complex voltage supervisors are interesting but also introduce a high-power consumption. The direct isolation of the microcontroller from the power supply is a better approach. The use of a voltage supervisor in conjunction with a MOSFET solves the problem of the high-power consumption in the reset state, which is the case in [98], where the proposed solution uses a semi-active circuit supervising the voltage and allowing the microcontroller input to stay enabled. This has the advantage that the microcontroller can switch itself off, after performing the necessary tasks. The circuit is able to perform a cold start from completely depleted storages, consumes much less power and prevents unnecessary energy usage. This solution is suitable for sensor nodes incorporating a microcontroller with a high off resistance.

Voltage supervisors will gain more importance in the IoT context, where a massive use of wireless sensors is intended, as the energy aspect becomes a big issue for usability as well as in applications where the energy availability is subject to large fluctuations.

### 4.6. Wake-Up Receiver for Sleeping/Idle Mode

**Figure 8:**
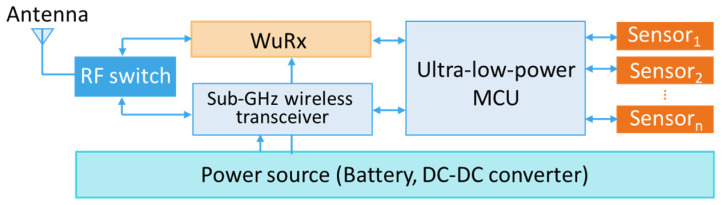
Block diagram of a typical wireless sensor node with a WuRx.

Using recent transceivers is usually associated to high power consumption during both transmit and receive phases. Some radio transceivers require an average of approximately 10 mA in receive mode and 20–30 mA in transmit mode for an ERP of 10–12 dBm. The use of those transceivers in energy autonomous WSNs is usually based on intermittent or duty cycling with certain transmitting, receiving and long sleep time intervals to save energy. During the sleep phase, the receiver is not active but also not completely switched off. Therefore, the system consumes power and latencies can massively increase. To satisfy the requirements regarding reaction time and low energy consumption, a separate so-called wake-up receiver (WuRx) (see Figure 8) with an ultra-low power consumption is required, allowing to switch the rest of the system completely off and have an always-on capability for on-demand communication with a power consumption in the µW range.

Wake-up receivers are used in addition to the main receiver, so that the main receiver is only activated when requested. As soon as a request signal with a unique identifier is received, it wakes up the main processor and other peripheral devices. The wake-up packet detection can be realized with suitable circuits having an ultra-low power consumption. Improving the sensitivity and increasing the bit rate for instance would inevitably lead to an increase in power consumption.

**Figure 9:**
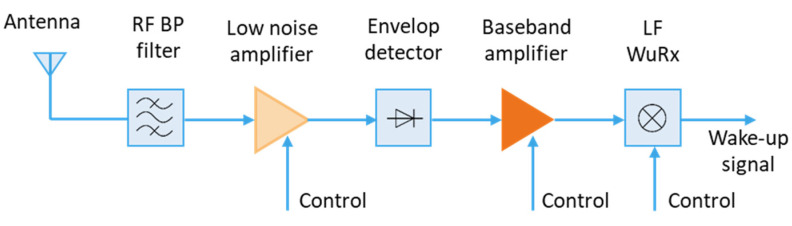
Block diagram of WuRx.

For decreasing the power consumption to an extreme low level, two main research approaches are followed. The first one focuses on developing new integrated circuits, allowing the optimization of power consumption in different frequency bands [99]. In the second one, off-the-shelf components are used to improve the time-to-market. For short range applications, different architectures based on passive components (see Figure 9) provide sensitivity improvements without a major increase in power consumption.

In [100], a passive architecture consisting of an envelope detector based on Schottky diode followed with a 16-bit D-Flip-Flop serial detector is introduced. The achieved sensitivity is by −52 dBm with a power consumption of 45 μW at a voltage of 3 V. The latency of the detection is in the range of 80 μs with a bit rate of 100 kbit/s. In [101], a WuRx is introduced based on a passive front-end, with a digital baseband consumption of 1.2 μW when monitoring a wake-up packet (WuPt). The minimum input power needed for a successful detection is by −55 dBm. A more sensitive WuRx is introduced in [102,103], which consumes 7.5 μW at a voltage of 3 V with a sensitivity of −60 dBm. The achieved latency is in the range of 10 ms with a bit rate of 4.56 kbit/s.

The performance of the mentioned designs is generally limited by the Schottky diode noise figure for detecting OOK-modulated (On-Off-Keying) signals [103]. Other concerned works enhance the transmission power efficiency of the WuPt. In [104], power-optimized waves are used to carry WuPt data and to increase the radio frequency to direct current (RF-DC) conversion efficiency of the envelope detector. This increases the rectified peak voltage while holding the incident wave’s average power constant. For long-range applications, a WuRx with higher sensitivity is required. For this reason, a low-noise amplifier (LNA) is generally added to boost the incoming signal to be properly detected by an envelope detector. Those architectures are known as tuned RF (TRF). Increasing the sensitivity based on a large gain would affect the noise figure of the following envelope detector. This leads to a significant amount of power. Therefore, smart duty cycling is generally used without increasing the intended short latency. In [105], a duty-cycle of 0.6% is applied on the TRF-based WuRx. It consumes an average power of 8.5 μW for a WuPt detection latency of 8.1 ms. The overall sensitivity is −73 dBm. In [106], a TRF duty cycled WuRx is presented with a sensitivity in the range of −90 dBm. The achieved latency of 32 ms is used for the detection of a 64-bit wake-up packet with a bit rate of 128 kbit/s. The power consumption is in the range of 3 μW.

Improving the sensitivity can also be realized with architectures allowing a better filtering of signals, e.g., super-heterodyne (SH) or super regenerative (SR) architectures. The authors of [107] introduced a SR-based WuRx with a power consumption of 40 μW and a sensitivity of −97 dBm. The decoding mechanism is performed on an off-the-shelf complex programmable logic device (CPLD), requiring much higher power than most recently published WuRx decoders.

## 5. Energy Saving on Network Level

Wireless communication consumes a big part of node energy for both the sending mode and the listening mode depending on the sending distance and the number of necessary hops to reach the sink. In this section, we provide a comprehensive review of the energy saving techniques at network level aiming to maintain the network with maximum node life. We provide an overview of methods and refer to several studies that we judge important and relevant, providing the references to help retrieve more details.

**Figure 10:**
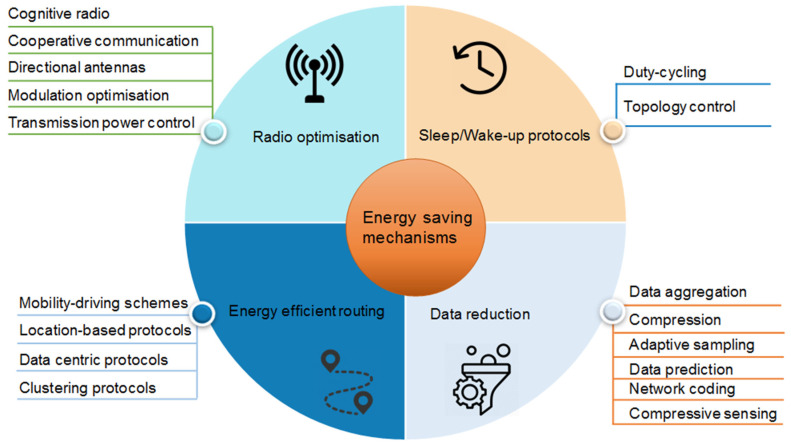
Energy saving mechanisms in wireless sensor networks.

Many energy saving techniques can reduce the energy consumption of nodes significantly and lower the requirements for the energy harvesting supply. They can increase the whole network life with a relatively high system reliability at low costs. Energy saving techniques can be classified into four main techniques: radio optimization, sleep/wake-up schemes, energy efficient routing protocols and data reduction (see Figure 10).

### 5.1. Radio Optimization

The radio module is the most energy consuming element for a sensor node. To decrease the overpower consumption during wireless communication, it is important to optimize the radio parameters by applying cognitive radio and cooperative communications techniques, optimizing antenna direction, modulation, and power transmission [108]. Cognitive radio (CR) techniques are used to reduce the power consumption for packet retransmission, resultant from packet losses, by alternating the access to multiple channels opportunistically. Sensor nodes can choose a communication channel in the wireless spectrum automatically and adjust the transmission and reception parameters according to channel conditions [109]. The authors of [110] showed that using CR improve the network lifetime by 13% when compared to the one without CR.

Cooperative communication techniques aim to significantly enhance the quality of the received signal using several single-antenna systems working together to form a virtual multi-antenna transmitter [111]. The importance of these techniques is the use of relay nodes as intermediate nodes, whereby the energy consumption is proportional to the square of the distance; therefore, decreasing the distance between the source and the destination reduces the energy considerably.

Directional antennas contribute to energy conservation by reducing the interference and data retransmission created by omnidirectional antennas [112]. While a node transmits data in a specific direction, a nearby node can also transmit at the same time without interference, thus saving a significant amount of energy. In the case a node intends to send a data packet to a neighbor node, it only sends in the specified direction, thus enhancing energy efficiency. However, using directional antennas requires finding the appropriate direction and parameters.

Modulation optimization techniques are interesting for determining the optimal modulation parameters of the radio with minimal power consumption [113]. Within short ranges, the circuit power consumption is higher than the consumed power for the transmission, which is the opposite for long distances. For this reason, it is important to set the appropriate modulation parameters that establish a trade-off between the transmission signal power and distance between nodes.

Transmission power control (TPC) is used to improve the energy by adjusting the radio transmission power according to some link quality parameters such as the link quality indicator (LQI) and the received signal strength indicator (RSSI) [114]. By reducing the transmission power, the interference is decreasing. In addition, higher transmission power allows the physical layer to incorporate the modulation at a higher bit/baud ratio. This can maximize the bandwidth in the case of heavy traffic. However, the delay in data transmission is significantly increased as more hops are involved to send a data packet. TPC protocols dissipate high energy during the initialization process.

### 5.2. Sleep/Wake-Up Protocols

Sleep/wake-up protocols offer a significant contribution to energy conservation by adapting the node activity. The basic idea of sleep/wake-up protocols is to put the radio of a wireless sensor node into a sleep state during the period of inactivity and wake it up just before transmitting or receiving a packet. Each transmitter node must know the sleep/wake schedule of the receiver node to communicate. Therefore, synchronization between the transmitter and receiver nodes is needed to enable them to wake-up at the same time. This can be carried out by applying different schemes including duty cycle and topology control. Duty cycling schemes aim to save energy by reducing the idle listening state and promoting the sleep mode. These schemes turn off the radio of a node most of the time and wake it up only if needed or at scheduled moments [115,116]. However, data latency issue occurs because the transmitter node needs to wait for the receiver node to be awake. To alleviate this problem, some parameters need to be settled, such as the time slot for each node, both idle listening and sleep periods and the length of the preamble.

The topology control techniques reduce the energy consumption by inactivating the nodes that are not needed for ensuring the coverage or connectivity. These techniques can be divided into location-driven and connectivity-driven approaches [117]. The location-driven approach determines the state of the node as either in active or in sleep mode and assigns which node to wake up and when depending on its location, which is known previously. The connectivity-driven approach is used to activate and deactivate dynamically a sensor node to ensure a full coverage and maintain the network connectivity.

### 5.3. Energy Efficient Routing

Routing protocols are important in wireless sensor networks to ensure end-to-end connectivity especially over long distances [118,119]. A small sending range is important for reducing energy consumption and limiting sending power. Energy efficient routing protocols reduce the energy consumption further by collecting data and selecting the energy saving path between the destination and source nodes. One possibility for aggregating the data in a network is via a mobile node [120], which collects the data from the normal nodes and transmits them to the base station (BS). The so-called mobility driven schemes present an efficient technique for minimizing the energy consumption by avoiding multi-hop communication and reducing the overload for the nodes closer to the base station, which typically occurs in other routing protocols [121]. In this energy saving technique, the mobile node needs to have a high energy budget, but the consumed energy in normal nodes is uniformly distributed. This technique is suitable in applications where regular data collection in a certain interval is taking place, such as walk-by solutions in smart metering or in robotics applications. However, in the case of large-scale networks, the mobile node takes a long time to collect data from different nodes, resulting in a considerable delay in data transmission. Thus, this technique is suitable for small-scale networks.

Depending on the design constraints for the network structure, other routing protocols can be adopted, such as data centric routing (flat), clustering-based routing (hierarchical) and location-based (geographic) routing [122]. Location-based routing protocols, also known as geographic routing protocols, use the information about the physical location of nodes provided by the Global Positioning System (GPS) [123] or some other localization techniques [72]. To reduce the energy consumption, a relay node is selected by the source node to forward the data packet toward the destination node. To this end, the source node determines the distance from neighbor nodes using different localization techniques such as RSSI [114].

Data centric routing protocols have a flat hierarchy [124], where all nodes have the same role and transmit data packets to a sink. At the beginning, the sink node sends queries to nodes within the deployment region and waits for incoming data. The intermediate nodes aggregates data from multiple sources and forwards the collected data towards the sink. Thereby, redundant data can be avoided, and energy can thus be saved.

Clustering-based hierarchical routing is an energy-efficient communication protocol based on three kinds of nodes: normal nodes (NN), cluster head (CH) and BS. NN sense the environment, get data and forward them to their associate CH, which in turn aggregates data from all cluster members and transmits them to the BS or, in a multi-hop network, to other CHs that take responsibility for transmission to the BS. Cluster-based hierarchical routing is one of the most interesting energy-aware techniques that minimizes simultaneously the number of transmitted messages and the distances to be covered between nodes. In addition, clustering may include data aggregation and fusion techniques, which reduce the overload as well as the packet losses [125].

### 5.4. Data Reduction

Data reduction contributes significantly to energy saving by reducing the packets size and therefore the sending time. This can be carried out by several methods, such as data aggregation, adaptive sampling, data prediction, network coding and compressive sensing [126]. In the data aggregation method, nodes collecting data, such as CH, conduct data fusion technique to decrease the overall amount of transmitted data, reducing data traffic and thus transmission delays.

Data compression techniques aim to reduce the data packet size by the node itself or by an aggregator node. By applying a data compression algorithm, such as Kalman filter, lossless compression and adaptive model selection [127,128,129,130], the data packet size can be significantly reduced. However, depending on the adopted algorithm, the data may be not accurately recovered, which results in a loss of precision.

Data prediction techniques intend to reduce the communication between nodes by preventing the transmission of each raw sample from source nodes to the sink node [131,132]. Therefore, the data traffic is decreased. For this purpose, each node creates a model to estimate the sensed data based on some observed values. This model is transmitted to the sink node to estimate upcoming data within an error tolerance. A new model is forwarded to the sink node only when a difference between predicted and observed values is detected by the source node. In some applications, where data precision is very important, such as in healthcare or military applications, this technique cannot be applied.

The adaptive sampling approach minimizes the amount of data packets using spatial or temporal correlation [133]. This technique is mostly used in centralized networks, where the sink node needs a high computational effort to process all the received data from different sources.

Network coding (NC) techniques aim to reduce the data traffic by allowing intermediate nodes between the source node and the sink to broadcast a binary or linear combination of multiple packets rather than forwarding a copy of each packet [134].

Compressive sensing (CS) techniques help significantly to save the energy by reducing the transmitted data volume [135]. With CS, the signal is transformed to a new signal with fewer values (sparsity). By reducing the sampling frequency, the energy consumption is decreased. A major feature of the CS is its potential to reconstruct a sparse signal from a limited number of measurements with no prior knowledge of the signal structure [135].

In this section, we review different energy saving techniques at the network level [13]. Radio optimization techniques reduce the signal quality to conserve the power supply. Energy-efficient routing protocols and sleep/wake-up protocols directly affect the network latency. Data reduction approaches can influence the reliability of the gathered information. Although recharging techniques for sensor nodes are highly relevant, energy-saving techniques are still crucial. The choice of a certain technique remains dependent on the specific scenario including the distances to be bridged, the possibility of fading due to obstacles, the number and distribution of nodes, the amount of data to be transmitted, the necessary measurement intervals and the possibility to harvest enough energy from ambient, which is decisive for the available energy budget. On the one hand, the available energy budget defines the necessary energy saving techniques to be applied. On the other hand, the energy saving can significantly reduce the size of the energy harvester and complexity of the energy management circuits and therefore the system costs.

## 6. Conclusions and Future Prospects

This review paper summarizes the main aspects to consider in the design of autonomous wireless sensor networks. An overview about possibilities of energy supply for WSNs including different solutions developed for energy transfer and energy harvesting from ambient sources is provided and discussed. Thereby, we highlight the concept of designing hybrid converters solutions in terms of multi-harvester and multi-sources. Energy supply through wireless charging also has several challenges which are illustrated.

In fact, not only the energy supply is needed but energy saving at the network level should be considered within the network design as well, which is discussed in this review paper in the last section. To overcome these high challenges, an interlocked interaction between different specialists should take place including material science, physics, system design, electronics and communication to realize necessary novel solutions.

In the future, based on novel developments in the fields of system design as well as semiconductors and microelectronic, we can expect that a battery-free WSN will have much more functionality with ultra-low energy budgets. This includes the sensors, their interfaces as well as signal processing and communication circuits. From another perspective, thanks to significant developments of material science, more efficient energy harvesters can be developed and can be better integrated into systems and structures. For example, flexible nanogenerators can be built using nanomaterials and polymers [136,137], 3D structures [138] or fiber for an improved integrability in textiles for wearables [139]. These are not the only perspectives. In addition, energy management concepts and components are evolving towards more intelligence and less energy consumption. In a few investigations, harvester and energy management can be coupled together such that control signals for the energy management are realized by the harvester, e.g., directly from the mechanical vibration of the source by a mechanical switch [79].

## Figures and Tables

**Table 1:** Selection of energy sources for supplying wireless sensors.

Sources	Harvesting Methods	Power Density	Characteristics	Availability
Solar [17]	Photovoltaic	15 mW/cm^2^	Performance depending on the solar cell technology and on environmental conditions. Maximum power point tracking (MPPT) is needed.	Outdoor
		<10 µW/cm^2^		Indoor
Wind [20]	Electromechanical conversion	0.1–6 mW/m^3^	Impedance matching needed to achieve a good energy yield.	Outdoor
Vibration [6,14,15,18,20,21]	Piezoelectric/ electromagnetic/ electrostatic/ magnetoelectric conversion/ triboelectric vibration	0.1–300 mW/cm^3^	Performance depends widely in the vibration source properties (frequency, frequency bandwidth). Rectifier is needed.	Indoor/ Outdoor
Thermal [19]	Thermoelectric conversion	40 µW/cm^3^	Application needs to realize a sufficient temperature gradient. Impedance matching needed	Indoor/ Outdoor
Ocean waves [34]	Piezoelectric conversion Triboelectric conversion	0.4–2 W/m^2^ 12 W/m^3^		Outdoor
Acoustic noise [35]	Piezoelectric conversion Electromagnetic Conversion Triboelectric Conversion	96 mW/cm^3^		Indoor/ Outdoor
RF [9]	Electromagnetic conversion	0.1 µW/cm^2^ (GSM 900 MHz) 1 µW/cm^2^ (WiFi)	Impedance matching needed	

**Table 2:** Drained current of different wireless sensor node for different activity modes.

Wireless Node Operation Mode	Panstamp NRG 2.0 [72,73]	Decawave DWM1000 [74]	9XTend [75]
**Off**	5 μA	-	5 μA
**Idle**	-	13.4 mA	147 μA
**Sleep**	1–2 μA	200–550 nA	1.6–10 mA
**Transmission**	36 mA (868 MHz)	111 mA (3.5 GHz)	-
**Reception**	18 mA (868 MHz)	154 mA (3.5 GHz)	-

## Data Availability

The data presented in this study are available on request from the corresponding author.
